# Cross-Presentation of a Spread-Defective MCMV Is Sufficient to Prime the Majority of Virus-Specific CD8+ T Cells

**DOI:** 10.1371/journal.pone.0009681

**Published:** 2010-03-12

**Authors:** Christopher M. Snyder, Jane E. Allan, Elizabeth L. Bonnett, Carmen M. Doom, Ann B. Hill

**Affiliations:** 1 Department of Molecular Microbiology and Immunology, Oregon Health and Science University, Portland, Oregon, United States of America; 2 School of Medicine and Pharmacology, The University of Western Australia, Crawley, Western Australia, Australia; Federal University of São Paulo, Brazil

## Abstract

CD8+ T cells can be primed by peptides derived from endogenous proteins (direct presentation), or exogenously acquired protein (cross-presentation). However, the relative ability of these two pathways to prime CD8+ T cells during a viral infection remains controversial. Cytomegaloviruses (CMVs) can infect professional antigen presenting cells (APCs), including dendritic cells, thus providing peptides for direct presentation. However, the viral immune evasion genes profoundly impair recognition of infected cells by CD8+ T cells. Nevertheless, CMV infection elicits a very strong CD8+ T cell response, prompting its recent use as a vaccine vector. We have shown previously that deleting the immune evasion genes from murine cytomegalovirus (MCMV) that target class I MHC presentation, has no impact on the size or breadth of the CD8+ T cell response elicited by infection, suggesting that the majority of MCMV-specific CD8+ T cells *in vivo* are not directly primed by infected professional APCs. Here we use a novel spread-defective mutant of MCMV, lacking the essential glycoprotein gL, to show that cross-presentation alone can account for the majority of MCMV-specific CD8+ T cell responses to the virus. Our data support the conclusion that cross-presentation is the primary mode of antigen presentation by which CD8+ T cells are primed during MCMV infection.

## Introduction

CD8+ T cells can be primed by endogenous antigen from proteins synthesized within professional APCs (direct priming), or by exogenous antigens engulfed by professional APCs (cross-priming) [1]. Cross-presentation has presumably evolved to ensure that a CD8+ T cell response is generated to viruses that cannot infect professional APCs, or which disable the ability of infected APCs to present antigen. There is abundant evidence that cross-presented antigen can prime robust CD8+ T cell responses [2], [3]. However, the role of cross-priming in a natural virus infection remains controversial [4].

Cytomegaloviruses (CMVs) are large beta herpesviruses expressing approximately 200 genes. Infection of both mice and humans with CMV elicits a broad CD8+ T cell response specific for an array of viral antigens [5]–[7]. In C57BL/6 (B6) mice acutely infected with murine cytomegalovirus (MCMV), CD8+ T cells specific for up to 26 different peptides from 19 viral proteins can be detected directly *ex vivo* by intracellular cytokine stimulation [6]. Moreover, the immunodominance hierarchy - the rank order of the strength of the response to the different epitopes - is consistent from animal to animal. The breadth of the response to MCMV in the B6 mouse provides an excellent tool with which to probe the nature of CD8+ T cell priming.

MCMV infects professional APCs, including dendritic cells *in vivo* [8], thus potentially providing antigen for direct presentation. However, it contains three immune evasion genes that impact the MHC class I pathway and profoundly impair CD8+ T cell recognition of infected cells [9]. Previously, we tested viruses that contained or lacked these three immune evasion genes to determine how the CD8+ T cell response would be influenced. Surprisingly, the immunodominance hierarchy was identical, for all 26 epitopes, whether or not the virus contained the immune evasion genes [10]. We interpreted these results to mean that the mode of priming during MCMV infection was identical for viruses with or without immune evasion genes. Either direct priming could occur normally despite the presence of the immune evasion genes, or the response was primed by cross-presentation. In contrast, introducing class I MHC immune evasion genes from human CMV into vaccinia virus resulted in an overall reduction in the frequency of vaccinia-specific CD8+ T cells and an absence of some, but not all, CD8+ T cell responses [11]. Thus, immune evasion genes targeting MHC class I are at least capable of disrupting CD8+ T cell priming and immunodominance in some circumstances. Based on all of these results, it seemed inconceivable that a profound inhibition of endogenous antigen presentation would not affect T cell priming to at least some MCMV antigens if direct presentation were involved. Thus, we hypothesized that the CMV-specific T cell responses were entirely elicited by cross-presented antigen.

Here we investigated the immune response to MCMV elicited when only cross-presentation is possible. Using a spread-defective MCMV, we demonstrate that cross-presentation of viral antigen produced by class I MHC-deficient fibroblasts is sufficient to prime a wide array of MCMV-specific CD8+ T cell responses, and generates a very similar immunodominance hierarchy to infection with wild-type virus. These results support the interpretation that cross-presentation is responsible for most CD8+ T cell priming in MCMV infection.

## Results and Discussion

In order to limit antigen presentation to the cross-presentation pathway, we needed to infect non-antigen-presenting cells with a virus that was incapable of spreading to infect other cells within the mouse, but which still produced all immunogenic viral proteins. Thus we focused on the viral glycoprotein L (gL). In all herpesviruses, gL forms a heterodimer with the glycoprotein H (gH) which, along with glycoprotein B, initiates membrane fusion and viral entry into the host cell (reviewed in [12]). Biochemical and mutagenesis studies of human CMV indicate that gL is an essential protein for beta herpesviruses [13]–[16]. Importantly, without the gL, CMV-gH is retained in the ER [17]. Thus, a virus lacking gL will also lack gH in the viral envelope.

**Figure 1:**
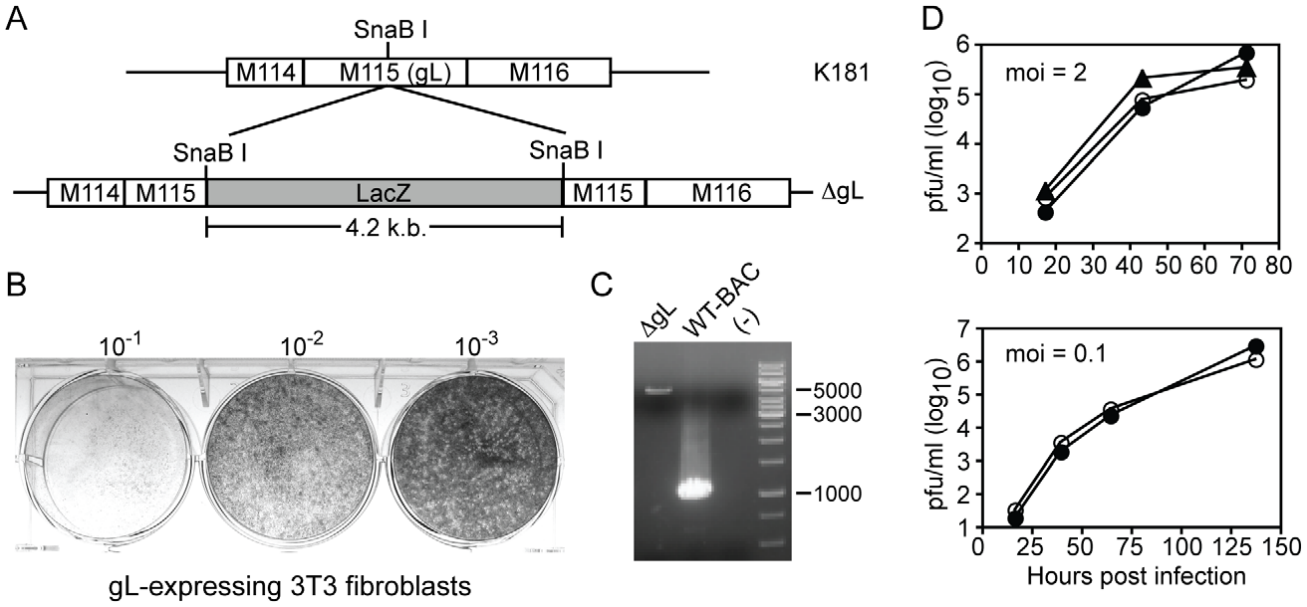
Deletion of glycoprotein L from MCMV can be complemented by gL-expressing cell line. A) Schematic of the strategy to functionally delete gL from the MCMV genome. B) ΔgL virus produced plaques on complementing cells. ΔgL virus was used to infect gL-expressing NIH-3T3 at the indicated dilutions and plaques were revealed at 10^−2^ and 10^−3^ dilutions by crystal violet staining 6 days later. C) The gL gene was detected by PCR in DNA extracted from wild-type or ΔgL viral preparations. D) The ΔgL virus grows with similar kinetics as the wild-type K181 virus in complementing cells. gL-3T3 were infected with the indicated virus at an moi = 2 or moi = 0.1. Cells were harvested at the indicated times and the presence of infectious virus was measured by plaque assay. Filled circles: K181 virus infected gL-3T3. Open circles: ΔgL infected gL-3T3. Filled triangles: K181 infected Balb-3T3. Data represents an individual experiment performed in duplicate.

We disrupted gL in the K181-Perth strain of MCMV by inserting a LacZ cassette into the middle of the M115 open reading frame (ΔgL - Figure 1A). To complement the mutation, NIH-3T3 cells were stably transfected with the M115 open reading frame (gL-3T3). The ΔgL virus was grown on the complementing gL-3T3 and produced plaques in a standard plaque assay using these supporting cells (Figure 1B). PCR for the gL gene confirmed the presence of the insert within the virus recovered from these cells (expected size is 5318 b.p. for ΔgL and 1118 b.p. for wild-type virus - Figure 1C). In addition, the ΔgL virus grew with similar kinetics as wild-type virus after both low and high multiplicities of infection (moi) (Figure 1D).

**Figure 2:**
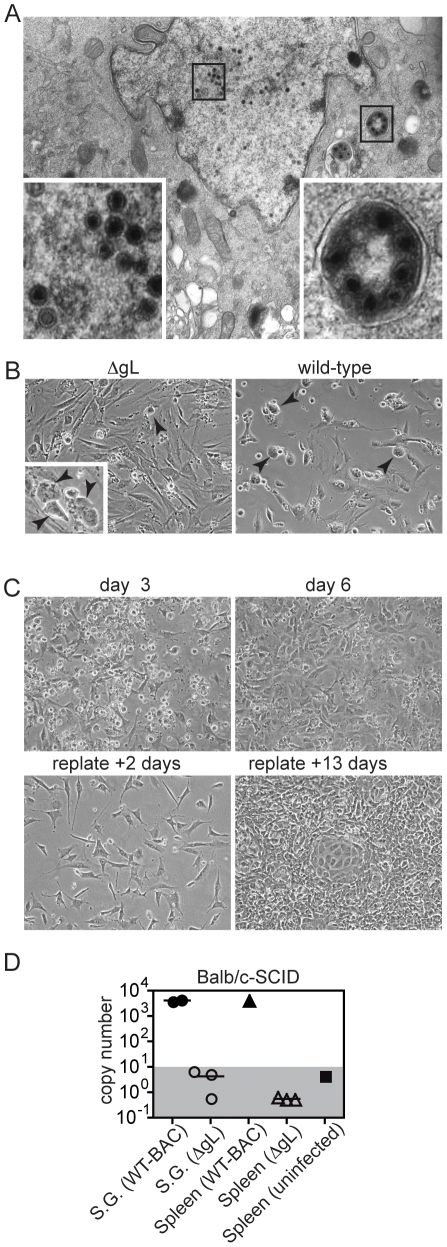
MCMV can not spread from cell to cell without the gL. A) Virions are produced in non-complementing cells. Non-complementing murine embryonic fibroblasts were infected with the ΔgL virus for 25 hours, fixed and prepared for electron microscopy. Images represent a magnification of 7100x. The insets are enlargements of the areas indicated by black boxes. B) The ΔgL virus does not spread in a culture of murine embryonic fibroblasts. Non-complementing murine embryonic fibroblasts (MEFs) were infected with wild-type or ΔgL virus at an moi = 0.3 and images of viral cytopathic effect were captured 3 days later. Images represent a magnification of 10x. Arrowheads indicate examples of cells displaying the typical cytopathic/rounding effect induced by MCMV infection. The inset in the left panel shows an enlarged image of the cytopathic/rounding effect in MEFs infected for 2 days with the ΔgL virus in an independent experiment. C) Non-complementing cells that survive infection can grow for at least 3 weeks. Non-complementing Balb-3T3 cells were infected with 1×10^5^ pfu (moi = 0.67) of the ΔgL virus. After 3 days rounded cells displaying the typical MCMV cytopathic effect are evident (upper left panel). After 6 days, the cells had grown to confluency (day 6 – upper right panel), were split into a larger volume containing all of the original supernatent and re-plated. Subsequent images were taken 2 days (lower left panel) and 13 days (lower right panel) after re-plating the ΔgL infected fibroblasts. Data are representative of 7 independent experiments. D) The ΔgL virus can not spread in immuno-compromised mice. Balb/c-SCID mice were infected i.p. with 1×10^5^ pfu of ΔgL or wild-type virus. 14 days after infection, DNA was extracted from the spleen and salivary gland (S.G.) and tested for the presence of MCMV by qPCR. Each symbol represents an individual mouse. Copies within the shaded area not distinguishable from background. Similar data was obtained in 2 other independent experiments.

Before testing whether cross-presentation could prime MCMV-specific CD8+ T cells, we needed to confirm that the ΔgL virus was completely spread defective. Since the gL-3T3 provide gL in *trans,* ΔgL virions grown on complementing cells should contain gL in the viral envelope but not the viral genome. Thus, our preparations of the ΔgL virus should be capable of a single round of infection in non-complementing cells. Indeed, nuclear, cytoplasmic and endocytic viral particles were evident by electron microscopy in infected non-complementing fibroblasts (Figure 2A) and cells displaying the typical MCMV-induced cytopathic/rounding effect were evident shortly after ΔgL infection (Figure 2B, left panel and inset). However, there was no evidence that the virus could spread beyond this initial round of infection. While a low dose of wild-type virus (moi = 0.3) infected most cells within 3 days of inoculation (Figure 2B, right panel), parallel cultures infected with the ΔgL virus grew more dense in this time period. In fact, the ΔgL virus never produced plaques on non-complementing cells and even when non-complementing cells were infected with over 1×10^5^ pfu of ΔgL (up to 3×10^5^ pfu in some experiments), the cells that survived the initial infection proliferated and were passaged for up to 4 weeks with no further evidence of viral growth (Figure 2C and data not shown). In contrast, mixing in as little as 3 pfu of wild-type K181 with 1×10^5^ pfu of ΔgL resulted in lysis of all fibroblasts in less than 2 weeks (not shown). These experiments demonstrated that the preparations of the ΔgL virus were unable to spread from cell to cell *in vitro* and did not contain a small, contaminating population of virus bearing a gL gene that had reverted. All of the stocks of ΔgL used in this study were confirmed to be spread defective *in vitro* in this manner. Nevertheless, to confirm that the ΔgL virus could not spread to other cells types *in vivo*, Balb/c-SCID mice were infected with 1×10^5^ pfu of wild-type or ΔgL virus. After two weeks, there was no evidence of viral DNA in either the spleens or salivary glands of mice infected with the ΔgL virus (Figure 2D). Thus, the ΔgL virus did not replicate and disseminate, even in immunodeficient mice. Together, these data confirm that the glycoprotein L is an essential glycoprotein for MCMV and that its deletion completely prevents viral spread.

**Figure 3:**
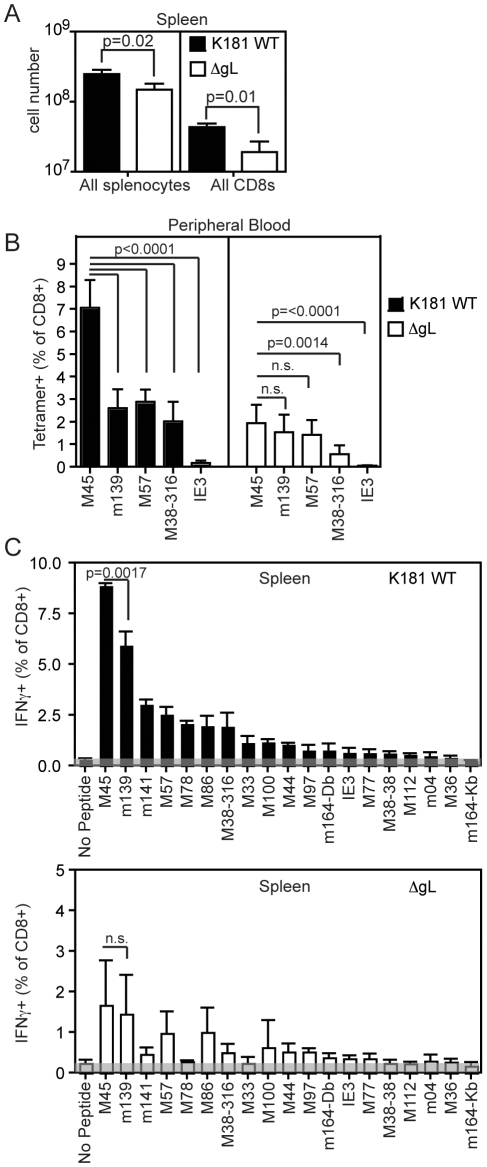
The ΔgL and K181 wild-type viruses induce a similar immunodominance hierarchy after direct i.p. infection. A) There are fewer splenocytes in ΔgL infected mice. C57BL/6 mice were infected i.p. with 1×10^5^ pfu K181 or ΔgL virus. 7 days after infection the total number of splenocytes and CD8+ T cells were quantified. Statisitical significance was determined by a Student's t-test. Data represents an average of 3 separate mice in this experiment. B) Immunodominant CD8+ T cell responses are readily detected in the blood of ΔgL infected mice. C57BL/6 mice were infected with the indicated viruses and the CD8+ T cell responses in the peripheral blood were examined by tetramer staining 7 days later (n = 7 mice per group combined from 2 independent experiments). Data is representative of at least 5 independent experiments. C) The immunodominance hierarchies induced by wild-type and ΔgL infections are remarkably similar. C57BL/6 mice were infected i.p. with 1×10^5^ pfu of the indicated viruses and spleens were harvested 7 days later (n = 3 mice per group). Cells were stimulated with the indicated peptides and the production of IFN-γ was measured as described. The shaded area reflects the background from unstimulated cells in this experiment. Similar data was obtained in an independent experiment that included a more limited subset of MCMV peptides (not shown).

Typically, MCMV infection develops progressively, infecting a limited subset of cells in the first round before spreading to other cells, including splenic DCs [18]. Since the ΔgL virus is only capable of a single round of infection, the types of cells infected *in vivo* should be limited. As viral protein expression is influenced by the cell types and organs that are infected [19]–[21], we might expect some differences in the immunodominance hierarchy elicited by ΔgL and replication-competent K181 MCMV. Moreover, the immunodominance hierarchy was characterized previously using the Smith strain of MCMV [6], [22], which could elicit a different pattern of CD8+ T cell responses. Thus, in order to characterize the CD8+ T cell response to K181 (wild-type) and ΔgL viruses, C57BL/6 (B6) mice were infected via the intraperitoneal route with either virus. T cell responses were measured at the peak of expansion, 7 days later. It was noted that the intensity of the immune response was lower after ΔgL infection in comparison to wild-type K181 infection. Relative to K181, there were approximately 40% fewer total splenocytes after ΔgL infection and half the number of CD8+ T cells (Figure 3A). Nevertheless, the immunodominance hierarchy was similar after the two infections. In the peripheral blood, M45-, m139- and M57-specific CD8+ T cells dominated the immune response, although the relative intensity of the M45-specific T cell response was significantly reduced compared to m139- and M57-specific responses after ΔgL infection (Figure 3B). When splenoctyes were tested with an expanded panel of 18 epitopes, the overall hierarchy was very similar for K181 and ΔgL infections (Figure 3C) and closely resembled, though did not precisely match, the hierarchy elicited by Smith MCMV [6], [22]. Again, the relative intensity of the M45-specific T cell response was significantly reduced in ΔgL infected mice. Moreover, the responses to three epitopes, m141, M78 and M33, appeared reduced relative to M57-, M86- and M38_316_-specific responses in ΔgL infected mice, although these differences were not statistically significant. MCMV infects many cell types *in vivo*, including epithelial cells, endothelial cells, macrophages and dendrtic cells [18], [23]. We speculate that the slight differences in immunodominance could reflect the intensity of viral gene expression in the cells infected immediately after injection, which is all that is available to the ΔgL virus.

**Figure 4:**
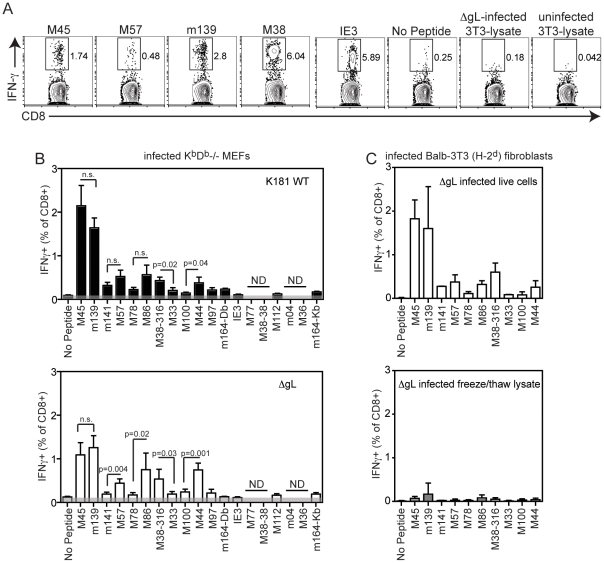
Cross-priming of antigen from fibroblasts infected with the ΔgL virus results in a similar immundominance hierarchy as wild-type infected fibroblasts. A) Balb-3T3s, which do not express H-2^b^ MHC and thus cannot directly stimulate CD8+ T cells from B6 mice, were left uninfected or infected with ΔgL virus for 3 hours. After infection, cells were washed with citrate buffer as described in the materials and methods and frozen immediately. The indicated peptides, representing a range of CD8+ T cell responses, or thawed lysate of infected cells (equivalent to the number of cells injected into each animal in B and C below) were used to stimulate splenic CD8+ T cells from a chronically infected C57BL/6 mouse and IFN-γ production was measured. B) K^b^D^b^-/- murine embryonic fibroblasts were infected with K181 or ΔgL viruses and washed as described. Infected cells were injected i.p. into C57BL/6 mice and IFN-γ production by T cells in the spleen was measured 7 days later after stimulation with the indicated peptides (n = 3 mice per group injected with wild-type infected cells and n = 4 mice per group injected with ΔgL infected cells in this experiment). ND  =  not done. The shaded area reflects the background from unstimulated cells in this experiment. Similar results were obtained from peripheral blood CD8+ T cells in an independent experiment. C) Balb-3T3s were infected with ΔgL and washed as above with a citrate buffer. After washing, cells were either left untreated or were frozen and thawed to produce a lysate. C57BL/6 mice were injected with live, ΔgL infected cells or the equivalent amount of frozen and thawed lysate from the same infection. 7 days later, splenic CD8+ T cells were stimulated with the indicated peptides and IFN-γ production was measured.

To investigate the CD8+ T cell response when priming was limited to cross-presentation, we infected class I MHC-deficient (K^b^D^b^-/-) MEFs *in vitro* with either wild-type K181 or ΔgL virus. Since these cells could not complement the missing gL protein, any virions produced by ΔgL-infected MEFs were non-infectious. Three hours after infection, the cells were washed with a citrate buffer to remove any infectious virions from the plasma membrane that were not yet internalized. No infectious virus from the inoculum was left in the culture after citrate washing as assessed by plaque assay, showing that the inoculum itself was unable to directly infect cells (data not shown). More importantly, cells treated in this manner, but frozen immediately after the citrate wash (before virions could be produced), were unable to stimulate CD8+ T cells harvested from chronically infected mice (Figure 4A), demonstrating a lack of antigenic peptides in the inoculum. Thus, citrate washed, infected cells were injected i.p. into B6 mice with the expectation that they would subsequently produce infectious (wild-type K181) or non-infectious (ΔgL) virions after transfer. Since the transferred K^b^D^b^-/- MEFs lacked any class I MHC molecules to present viral antigen, mice receiving ΔgL infected fibroblasts should only mount a CD8+ T cell response if endogenous cells can capture and cross-present viral antigen in class I MHC. As shown in Figure 4B, 7 days after fibroblast transfer, ΔgL infected fibroblasts were able to prime the majority of the tested MCMV-specific CD8+ T cell responses. In fact, the immunodominance hierarchy was strikingly similar between mice that received wild-type K181 or ΔgL infected MEFs. Inoculating mice with infected fibroblasts resulted in a reduced relative intensity of M45-specific T cells in all mice and a reduction in the relative immunodominance of m141-, M78- and M33-specific T cells, which was now statistically significant in ΔgL-primed mice (compare Figure 4B and C with Figure 3C). Moreover, the immunodominance of M100-specific T cells was reduced, while M44-specific T cells remained easily detectible. It is notable that the immunodominance hierarchy was similar between mice that received wild-type infected or ΔgL-infected fibroblasts, but slightly different from mice that were directly infected with wild-type virus (compare Figure 4B with Figure 3C). Again, this may reflect the fact that viral proteins are differentially expressed in different cell types [19]–[21]. However, direct infection with the ΔgL virus, where infection of DCs for direct presentation can occur, elicited a response that is very similar to that elicited by ΔgL-infected fibroblasts, where only cross-presentation is possible. This slightly altered immunodominance was reproduced by injecting ΔgL-infected Balb-3T3 fibroblasts, which express H-2^d^ MHC and thus can not directly stimulate CD8+ T cells from B6 mice (Figure 4C). In contrast, lysate produced by freezing these infected cells immediately after the citrate wash resulted in weak or undetectable CD8+ T cell responses to all tested antigens, again indicating that nothing in the inoculum itself was able to directly prime CD8+ T cell responses *in vivo* (Figure 4C). Overall, these data show that cross-presented viral antigen was sufficient to prime the majority of CD8+ T cell responses seen after MCMV infection.

Our data support the conclusion that cross-presentation is the primary mode of antigen presentation by which CD8+ T cells are primed during MCMV infection. Since long-lived antigens are much more likely to be cross-presented than short-lived antigens [24], [25], our results imply that the CD8+ T cell response during CMV infection is limited by the kinetics of protein decay. The recent impressive results obtained using CMV as a vaccine vector for SIV [26] will likely spark interest in its use as a vaccine vector for other diseases. Our data suggest that long-lived proteins may be more suitable for use in a CMV-based vaccine than short-lived proteins.

We do not expect that CD8+ T cell priming during most virus infections will be limited to cross-presented antigen. Indeed, intravital microscopy has observed naïve CD8+ T cells interacting with directly vaccinia-infected DCs and upregulating the early activation marker CD69 [27]. If T cell priming in MCMV infection is exclusively reliant on cross-presentation, it cannot be solely due to the actions of the immune evasion genes that attack the direct presentation pathway, since removing those genes did not alter the immunodominance hierarchy. Rather, we postulate that other MCMV genes that affect DC function are responsible for the absence of direct priming. Studies of *in vitro* and *ex-vivo* MCMV-infected DCs have shown that infected DCs downregulate the costimulatory molecules CD80, CD86 and CD40 and are unable to prime naïve CD8+ T cells [8], [28]. Moreover, Benedict and colleagues have recently demonstrated that infected DCs upregulate the inhibitory ligands PD-L1 and PD-L2, and that the PD-L1/PD-1 interaction is important for inhibiting T cell expansion [29]. Thus, it may be that modulation of the function and maturation of infected DCs, rather than inhibition of antigen presentation *per se*, determines the relative importance of direct *versus* cross-priming of CD8+ T cells in MCMV infection. If the viral genes controlling these functions can be deleted, it might be possible to allow direct priming and potentially alter the immunodominance elicited by MCMV infection.

## Materials and Methods

### Viruses

The ΔgL virus was produced from the K181-Perth strain of MCMV, through insertion of a *lacZ* expression cassette under the control of the HCMV IE promoter by homologous recombination into the gL ORF as described elsewhere [(Allan et. al., manuscript submitted) and [30]]. To confirm that the LacZ gene was inserted into M115 (gL), viral DNA was subjected to PCR using primers that annealed to the surrounding genes M114 and M116 (M114 to M115: TGCGTGGCGGATAGTTTCAG; M116 to M115: AACGACTCGCTCATTATCG. Stocks of the ΔgL virus were produced on gL-3T3 cells (see below). ΔgL virus was compared to the K181 strain of MCMV or the MCMV strain MW97.01, which is derived from a bacterial artificial chromosome of the Smith strain [31]. Plaque assays were performed without centrifugal enhancement using gL-3T3 or Balb-3T3 cells to determine the number of plaque forming units (pfu) per ml.

### Cells

To produce a gL-expressing NIH-3T3 cell line (gL-3T3), contains the gL ORF amplified by PCR from K181 MCMV DNA as described elsewhere (Allan et. al. manuscript submitted). This cell line was grown in complete media supplemented with 500 µg/ml Geneticin (G-418, Gibco).

### Mice and infections

Balb/c-SCID mice (CBySmn.CB17-Prkdcscid/J) and C57BL/6 mice were purchased from Jackson Laboratories. KbDb-/- murine embryonic fibroblasts (MEFs) were derived from double-deficient mice [32] kindly provided by David Raulet and bred in house. SCID mice were infected i.p. with 1×10^5^ pfu of ΔgL or WT-BAC [31] and organs were harvested 2 weeks later. For direct infection of B6 mice, 1×10^5^ pfu of ΔgL or K181 virus was injected via the intraperitoneal route. For indirect infections, 1×10^5^ K^b^D^b^-/- MEFs or 2×10^5^ Balb/c-3T3 fibroblasts were plated overnight in 6 well plates before infection with 3×10^5^ pfu of ΔgL or K181 virus. After 3 hours, cells were washed twice with PBS, incubated for 60 seconds with 1 ml of citrate buffer (50 mM sodium citrate, 4 mM potassium chloride, pH = 3) and washed twice more with complete media (DMEM + 10% FetalPlex – Gemini Bioproducts). Cells then harvested with trypsin-EDTA (Gibco), washed and resuspended in PBS for injection. To confirm an absence of infectious virus or viral peptides, lysate from these cells was used in a standard plaque assay with gL-3T3s or to stimulate CD8+ T cells from chronically infected mice. B6 mice received approximately 2×10^5^ MEFs or Balb-3T3s by i.p. injection or equivalent amounts of lysate from frozen and thawed, infected cells.

### Quantitative PCR

Quantitative PCR for the MCMV E1 gene was performed using the Platinum qPCR Supermix (Invitrogen). The primers and probe were synthesized by Invitrogen and Applied Biosystems respectively. VIC labeled probe: ACTCGAGTCGGACGCTGCATCAGAAT; Primer E1 for: TCGCCCATCGTTTCGAGA; Primer E1 rev: TCTCGTAGGTCCACTGACGGA. To calculate the genome copy number, a standard curve of a plasmid containing the E1 gene from MCMV (pGEM-T-E1) was included in every assay.

### Intracellular cytokine stimulation assay

Analysis of intracellular IFN-γ was performed as previously described [6], [33], [34] using the indicated peptides (synthesized by Genemed Synthesis, Inc). Samples were acquired on an LSR II flow cytometer (BD Biosciences) and data were analyzed with FlowJo software (Tree Star).

### Ethics Statement

All animal work was approved by the Institutional Biosafety Committee and the Institutional Animal Care and Use Committee at OHSU.
