# Correction: Cross-presentation of a spread-defective MCMV is sufficient to prime the majority of virus-specific CD8+ T cells

**DOI:** 10.1371/journal.pone.0226705

**Published:** 2019-12-13

**Authors:** Christopher M. Snyder, Nicholas Davis-Poynter, Helen E. Farrell, Jane E. Allan, Elizabeth L. Bonnett, Carmen M. Doom, Ann B. Hill

Nicholas Davis-Poynter and Helen E. Farrell should be included in the author byline. Nicholas Davis-Poynter should be listed as the second author and Helen E. Farrell should be listed as the third author. The correct author affiliations are: Christopher M. Snyder^1^, Nicholas Davis-Poynter^2^, Helen E. Farrell^2^, Jane E. Allan^3^, Elizabeth L. Bonnett^1^, Carmen M. Doom^1^, Ann B. Hill^1^

**1** Department of Molecular Microbiology and Immunology, Oregon Health and Science University, Portland, Oregon, United States of America **2** Department of Microbiology, The University of Western Australia, Perth, Australia **3** School of Medicine and Pharmacology, The University of Western Australia, Crawley, Australia

The correct citation is: Snyder CM, Davis-Poynter N, Farrell HE, Allan JE, Bonnett EL, Doom CM, et al (2010) Cross-Presentation of a Spread-Defective MCMV Is Sufficient to Prime the Majority of Virus-Specific CD8+ T Cells. PLoS ONE 5(3): e9681. https://doi.org/10.1371/journal.pone.0009681
